# Quantitative anti-HBc combined with quantitative HBsAg can predict HBsAg clearance in sequential combination therapy with PEG-IFN-α in NA-suppressed chronic hepatitis B patients

**DOI:** 10.3389/fimmu.2022.894410

**Published:** 2022-07-26

**Authors:** Wen-Xin Wang, Rui Jia, Ying-Ying Gao, Jia-Ye Liu, Jun-Qing Luan, Fei Qiao, Li-Min Liu, Xiao-Ning Zhang, Fu-Sheng Wang, Junliang Fu

**Affiliations:** ^1^ Peking University 302 Clinical Medical School, Beijing, China; ^2^ Senior Department of Infectious Diseases, The Fifth Medical Center of Chinese People's Liberation Army (PLA) General Hospital, National Clinical Research Center for Infectious Diseases, Beijing, China; ^3^ Medical School of Chinese People's Liberation Army (PLA), Beijing, China

**Keywords:** chronic hepatitis B, anti-HBc, HBcrAg, HBsAg clearance, combined treatment, PEG-IFN-α

## Abstract

**Background and aims:**

Precise predictors are lacking for hepatitis B surface antigen (HBsAg) clearance under the combination therapy of nucleos(t)ide analogs (NA) and pegylated interferon-alpha (PEG-IFN-α) in patients with chronic hepatitis B (CHB). This study aimed to determine the quantitative anti-hepatitis B core antibody (qAnti-HBc) and quantitative hepatitis B core-related antigen (qHBcrAg) as predictors for HBsAg clearance in NA-suppressed patients with CHB receiving PEG-IFN-α add-on therapy.

**Methods:**

Seventy-four CHB patients who achieved HBV DNA suppression (HBV DNA < 20 IU/ml) and quantitative HBsAg (qHBsAg) < 1,500 IU/ml after ≥1 year of NA treatment were enrolled. Fifteen patients continued on NA monotherapy, while 59 patients received PEG-IFN-α add-on therapy. Serum qAnti-HBc and qHBcrAg levels were detected every 12 or 24 weeks for add-on and NA-alone groups, respectively.

**Results:**

Serum qAnti-HBc but not qHBcrAg levels at baseline were negatively correlated with the duration of prior NA therapy. After 48-week treatment, both qAnti-HBc and qHBcrAg levels declined further, and 17/59 (28.81%) and 0/15 (0%) achieved HBsAg clearance in add-on and NA groups, respectively. In the add-on group, the rate of HBsAg clearance was significantly higher in patients with baseline qAnti-HBc < 0.1 IU/ml (52.63%). Logistic regression analysis identified baseline qAnti-HBc but not qHBcrAg, which was an independent predictor for HBsAg loss. Receiver operating characteristic curve analysis showed that the combination of qAnti-HBc and qHBsAg had a better predictive value for HBsAg clearance.

**Conclusions:**

A combination of qHBsAg and baseline qAnti-HBc levels may be a better prediction strategy for HBsAg clearance in NA-suppressed CHB patients receiving PEG-IFN-α add-on therapy.

## Introduction

Approximately 250 million individuals worldwide are chronically infected with hepatitis B virus (HBV) (1), and 10%–15% of them progress to chronic liver diseases, such as chronic hepatitis B (CHB), cirrhosis, and hepatocellular carcinoma (HCC) (2). Currently, achieving both HBV DNA undetectable and hepatitis B surface antigen (HBsAg) clearance with or without hepatitis B surface antibody (HBsAb) appearance, named functional cure, can significantly reduce the incidence of cirrhosis and HCC (3–5). However, the efficacy of available drugs, including nucleos(t)ide analogs (NA) and interferon (IFN), remains unsatisfactory when used alone. Recently developed new strategies of “add-on” or “switch-to” pegylated-interferon-alpha (PEG-IFN-α) to ongoing NA treatment have dramatically improved the chance of achieving sustained HBsAg loss/seroconversion in CHB patients (6–12). In patients with quantitative HBsAg (qHBsAg) < 1,500 IU/ml with or without early on-treatment HBsAg decline, the loss rate can reach 22.2%–58.7% after 48–96 weeks of therapy (6–10). Approximately 40%–80% of these patients remain HBsAg positive after suffering from side effects of PEG-IFN-α. Therefore, new indicators or strategies to precisely identify the patients who are most likely to achieve HBsAg clearance under PEG–IFN-α-based therapy are vital.

The quantitative anti-hepatitis B core antibody (qAnti-HBc) and quantitative hepatitis B core-related antigen (qHBcrAg) are emerging novel markers for patients with CHB. Studies have shown that the qAnti-HBc levels in CHB patients during the immune clearance and reactivation phases are significantly higher than those in the immune tolerance and low replication phases in natural history (13, 14). The qAnti-HBc levels are strongly correlated with serum ALT levels and the hepatic inflammatory degree in treatment-naïve patients (15–17) and showed a gradually decreasing trend in the course of NA therapy (18). HBcrAg levels are closely correlated with intrahepatic covalently closed circular DNA (cccDNA) transcriptional activity in CHB patients (19). Both qAnti-HBc and qHBcrAg levels could predict the clearance of hepatitis B e antigen (HBeAg) during naive treatment with PEG-IFN-α or NA therapy (16, 20–22). Moreover, the decline in qHBcrAg levels is also associated with persistent HBV DNA suppression after PEG-IFN-α-based treatment (23).

However, it remains unclear whether qAnti-HBc and qHBcrAg levels can predict HBsAg clearance in NA-suppressed CHB patients receiving combination therapy with PEG-IFN-α. This study aimed to investigate the kinetics of serum qAnti-HBc and qHBcrAg levels in CHB patients with qHBsAg < 1,500 IU/ml receiving PEG-IFN–α add-on to ongoing NA therapy, and to evaluate the role of these two biomarkers in predicting HBsAg clearance.

## Materials and methods

### Study population and design

**Figure 1:**
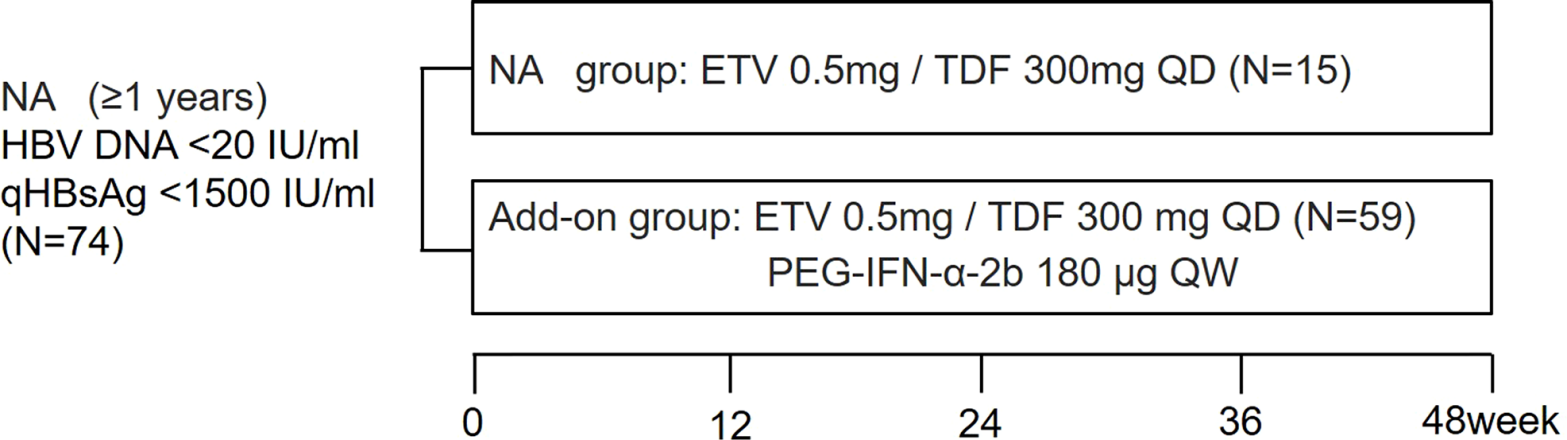
Study design. Enrolled patients were divided into NA and add-on groups according to their wishes. Blood samples were collected at study entry and every 24 weeks (NA group) or every 12 weeks (add-on group). NA, nucleos(t)ide analogs; qHBsAg: quantitative hepatitis B surface antigen; ETV: entecavir; TDF, tenofovir disoproxil fumarate; PEG-IFN-α-2b, Pegylated-interferon-alpha-2b; QD: once daily; QW: once a week.

The subjects were enrolled from the Fifth Medical Center of Chinese PLA General Hospital. CHB patients aged 18–65 years who had received NA treatment for ≥1 year and achieved serum qHBsAg < 1,500 IU/ml and HBV DNA suppressed were eligible. HBV DNA suppressed is defined as HBV DNA < 20 IU/ml. Patient exclusion criteria were as follows: coinfection with human immunodeficiency virus, hepatitis A virus, hepatitis C virus, hepatitis D virus, or hepatitis E virus; presence of other chronic liver disease or serious systemic diseases; and receiving IFN, glucocorticoids, or other immunomodulatory therapy within 6 months prior to enrollment. A total of 74 patients were enrolled and divided into a continuing NA monotherapy group (NA group, 15 patients) and an add-on PEG-IFN-α therapy group (add-on group, 59 patients) according to their wishes. The NA group received continuous entecavir (ETV) or tenofovir disoproxil fumarate (TDF) for 48 weeks. The add-on group received ETV or TDF combined with PEG-IFN-α-2b (180 μg once a week) for 48 weeks (**Figure 1**). The primary outcome was HBsAg loss after 48 weeks of treatment.

ETV was the main type of NA taken before enrollment (NA group: 10/66.67%; add-on group: 37/62.71%). If patients received lamivudine, adefovir dipivoxil, or telbivudine before enrollment, they were replaced with ETV or TDF. The baseline characteristics of the patients were comparable between the groups (**Table 1**). The study was approved by the Ethics Committee of the Fifth Medical Center of Chinese PLA General Hospital.

**Table 1:** Characteristics of the enrolled patients.

Indicators	NA group (*n* = 15)	Add-on group (*n* = 59)	*p*
Male/female, *n*	13/2	49/10	1.000
Age (years), median (quartiles)	38.0 (34.0, 47.5)	39.0 (32.0, 47.0)	0.968
Route of transmission, maternal/paternal/others, *n*	1/3/11	13/9/37	0.407
Duration of prior NA treatment (months), median (quartiles)	49.0 (24.0, 70.0)	63.0 (39.0, 121.0)	0.143
Baseline qHBsAg (log_10_IU/ml), median (quartiles)	2.73 (2.21, 3.01)	2.59 (1.97, 2.95)	0.550
Baseline HBeAg positive, *n* (%)	6 (40.00)	18 (30.51)	0.695
Baseline HBeAb positive, *n* (%)	7 (46.67)	25 (42.37)	0.764
Baseline qAnti-HBc (log_10_IU/ml), median (quartiles)	1.81 (0.12, 2.94)	1.97 (−1.00, 2.70)	0.618
Baseline qHBcrAg (log_10_U/ml), median (quartiles)	4.31 (3.25, 5.50)	4.22 (3.52, 5.16)	0.718
Baseline ALT (U/L), median (quartiles)	20.0 (14.0, 30.5)	22.0 (18.0, 27.0)	0.642
Baseline AST (U/L), median (quartiles)	25.0 (21.0, 29.0)	21.0 (19.0, 24.0)	0.107
HBsAg loss at week 48, *n* (%)	0 (0)	17 (28.81)	0.043

NA, nucleos(t)ide analogs; Add-on, nucleos(t)ide analogs combined with pegylated-interferon-alpha; HBsAg, hepatitis B surface antigen; qHBsAg, quantitative hepatitis B surface antigen; HBeAg, hepatitis B e antigen; HBeAb, hepatitis B e antibody; qAnti-HBc, quantitative anti-hepatitis B core antibody; qHBcrAg, quantitative hepatitis B core-related antigen; ALT, alanine aminotransferase; AST, aspartate amino transferase.

### Clinical and laboratory evaluation

Twenty milliliters of peripheral blood samples were collected at study entry and every 24 weeks (NA group), or every 12 weeks (add-on group). Serological and biochemical markers were measured routinely in a central clinical laboratory. Serum HBV DNA levels were tested using the COBAS AmpliPrep/COBAS TaqMan HBV Test (Roche Molecular Systems, Inc., Branchburg, USA); the lower limit of detection (LLD) of HBV DNA level was 20 IU/ml. Serum HBsAg levels were quantified using Elecsys HBsAg II quant II (Roche Diagnostics GmbH, Mannheim, Germany); the LLD of qHBsAg was 0.05 IU/ml. Other HBV markers, including HBsAb levels, HBeAg, and HBeAb, were detected using COBAS e602 (Roche Diagnostics GmbH, Mannheim, Germany). Serum qAnti-HBc levels were measured using a double–antigen sandwich immune assay (Wantai, Beijing, China), as reported previously (24). In detail, both high-value quantitative detection (HQ) and general quantitative detection (GQ) were used in our study. The detection range of the HQ kit and the GQ kit was 100–100,000 IU/ml and 0.1–25 IU/ml, respectively. If the test result of a sample was higher than the upper limit of the GQ kit and lower than the lower limit of the HQ kit, the sample would be diluted by five times and re-tested it with GQ kit. Thus, the LLD of the qAnti-HBc level was 0.1 IU/ml. Serum qHBcrAg levels were measured with a fully automated Lumipulse analyzer (Fujirebio, Tokyo, Japan) by chemiluminescent enzyme immunoassay with the detection limit 2.0 to 7.0 log_10_ U/ml. Serum qAnti-HBc and qHBcrAg levels were measured according to the manufacturer’s instructions.

### Statistical analysis

Serum qHBsAg, qAnti-HBc, and qHBcrAg levels were log-transformed. Statistical analyses were performed using SPSS 25.0 software (IBM Corp., Armonk, NY, USA). Continuous variables were expressed as median (quartiles). The statistical significance of the difference between two groups was determined using the Mann–Whitney *U* test. The Wilcoxon signed ranks test was used for comparison of two related samples. Categorical variables were analyzed using the chi-squared test. Pearson correlation was used to determine the correlation between continuous variables. The HBsAg loss rates between patients with qAnti-HBc < 0.1 IU/ml and >0.1 IU/ml were re-evaluated with propensity score matching (PSM), adjusting for demographic characteristics and baseline indicators associated with HBV infection. A PSM with the nearest-neighbor method using 1:2 matching was performed by R version 4.1.2. Univariate and multivariate logistic regression analyses were used to identify predictors for HBsAg clearance. The discriminatory power of various predictors for HBsAg clearance was tested with the area under the receiver operating characteristic (ROC) curve. All statistical analyses were based on two-tailed hypothesis tests with a significance level of p < 0.05.

## Results

### Serum qAnti-HBc and qHBcrAg levels were decreased during treatment

The levels of baseline serum qAnti-HBc and qHBcrAg in the NA group were slightly higher than in the add-on group but without significant difference (p > 0.05) (**Table 1**).

**Figure 2:**
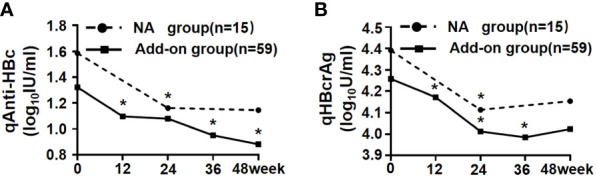
Kinetics of serum qAnti-HBc and qHBcrAg in NA and add-on groups. **(A)** Kinetics of qAnti-HBc in two groups. **(B)** Kinetics of qHBcrAg in two groups. * Comparison in biomarkers between this and last time points and *p* < 0.05. NA, Nucleos(t)ide analogs; qAnti-HBc, quantitative anti-hepatitis B core antibody; qHBcrAg, quantitative hepatitis B core-related antigen.

In the course of treatment after enrollment, serum qAnti-HBc and qHBcrAg levels in both add-on and NA groups were further declined, and there was no difference in the reduction between the groups [qAnti-HBc: 0.19 (0.00, 0.48) vs. 0.09 (0.00, 0.36) log_10_IU/ml, p = 0.334; qHBcrAg: 0.23 (0.00, 0.45) vs. 0.13 (0.00, 0.31) log_10_U/ml, p = 0.737] (**Figures 2A, B**). Further analysis of the reduction process of these two markers showed that serum qAnti-HBc and qHBcrAg levels in the add-on group had been maintained on a downward trend at almost every time point: qAnti-HBc levels declined significantly at weeks 12, 36, and 48 compared with the previous follow-up time point (p < 0.001, p = 0.012, and p = 0.008, respectively) (**Figure 2A**), and qHBcrAg declined significantly at weeks 12, 24, and 36 compared with the previous follow-up time point (p < 0.001, p < 0.001, and p = 0.031, respectively) (**Figure 2B**). In the NA group, qAnti-HBc and qHBcrAg levels decreased significantly only in the first 24 weeks of treatment (p = 0.047 and p = 0.002, respectively) (**Figures 2A, B**).

### Baseline serum qAnti-HBc levels can predict HBsAg clearance under add-on treatment

**Figure 3:**
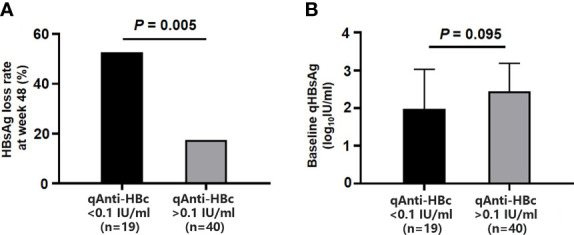
Comparison of HBsAg loss rates at week 48 and baseline qHBsAg in the add-on group with baseline qAnti-HBc <0.1 IU/ml and > 0.1 IU/ml. **(A)** Comparison of HBsAg loss rates at week 48 in two subgroups. **(B)** Comparison of baseline qHBsAg in two subgroups. HBsAg, hepatitis B surface antigen; qHBsAg, quantitative HBsAg; qAnti-HBc, quantitative anti-hepatitis B core antibody.

After 48-week treatment, 17/59 (28.81%) patients achieved HBsAg clearance in the add-on group, while no patients achieved HBsAg clearance in the NA group (**Table 1**). In the add-on group, the levels of baseline qAnti-HBc and qHBsAg in patients who achieved HBsAg clearance were significantly lower than those who did not, but baseline qHBcrAg levels were comparable between the two subsets of patients (**Table 2**). The rate of HBsAg clearance was significantly higher in patients with baseline qAnti-HBc < 0.1 IU/ml than in patients with baseline qAnti-HBc > 0.1 IU/ml (52.63% vs. 17.50%, p = 0.005) (**Figure 3A**). Meanwhile, baseline qHBsAg levels between the two groups show no significant difference (**Figure 3B**). We also compared gender, age, baseline qHBcrAg, duration of prior NA treatment before enrollment, baseline HBeAg positive rate, and baseline ALT between the two groups. We found no statistical differences (p > 0.05). A 1:2 PSM analysis was further conducted to avoid the influence of the above factors. After PSM, 18 patients from the baseline qAnti-HBc < 0.1 IU/ml group and 35 patients from the baseline qAnti-HBc > 0.1 IU/ml group were included in further analysis. The rate of HBsAg clearance in patients with baseline qAnti-HBc < 0.1 IU/ml and > 0.1 IU/ml was 50.00% and 20.00% (p = 0.024), respectively.

**Table 2:** Baseline characteristics of patients who achieved HBsAg clearance or not after 48-week add-on therapy.

Indicators	HBsAg clearance, *n* (%)		*p*
	No (*n* = 42)	Yes (*n* = 17)	
Male, *n* (%)	35 (83.33)	14 (82.35)	1.000
Age (years), median (quartiles)	39.5 (32.5, 47.0)	37.0 (31.0, 46.0)	0.592
Route of transmission, maternal/paternal/others, *n*	11/7/24	2/2/13	0.424
Duration of prior NA treatment (months), median (quartiles)	66.0 (38.0, 125.0)	51.0 (47.0, 78.0)	0.483
qHBsAg (log_10_IU/ml), median (quartiles)	2.74 (2.46, 3.05)	1.69 (0.83, 2.05)	<0.001
HBeAg positive, *n* (%)	12 (28.57)	6 (35.29)	0.612
HBeAb positive, *n* (%)	19 (45.24)	6 (35.29)	0.484
qAnti-HBc (log_10_IU/ml), median (quartiles)	2.11 (1.47, 2.77)	−1.00 (−1.00, 2.29)	0.031
qHBcrAg (log_10_U/ml), median (quartiles)	4.24 (3.45, 5.18)	4.15 (3.59, 5.07)	0.958
ALT (U/L), median (quartiles)	22.0 (18.0, 27.25)	20.0 (14.0, 27.0)	0.564
AST (U/L), median (quartiles)	21.0 (19.0, 24.25)	21.0 (20.0, 24.0)	0.909

NA, nucleos(t)ide analogs; Add-on, nucleos(t)ide analogs combined with pegylated-interferon-alpha; HBsAg, hepatitis B surface antigen; qHBsAg, quantitative hepatitis B surface antigen; HBeAg, hepatitis B e antigen; HBeAb, hepatitis B e antibody; qAnti-HBc, quantitative anti-hepatitis B core antibody; qHBcrAg, quantitative hepatitis B core-related antigen; ALT, alanine aminotransferase; AST, aspartate amino transferase.

**Figure 4:**
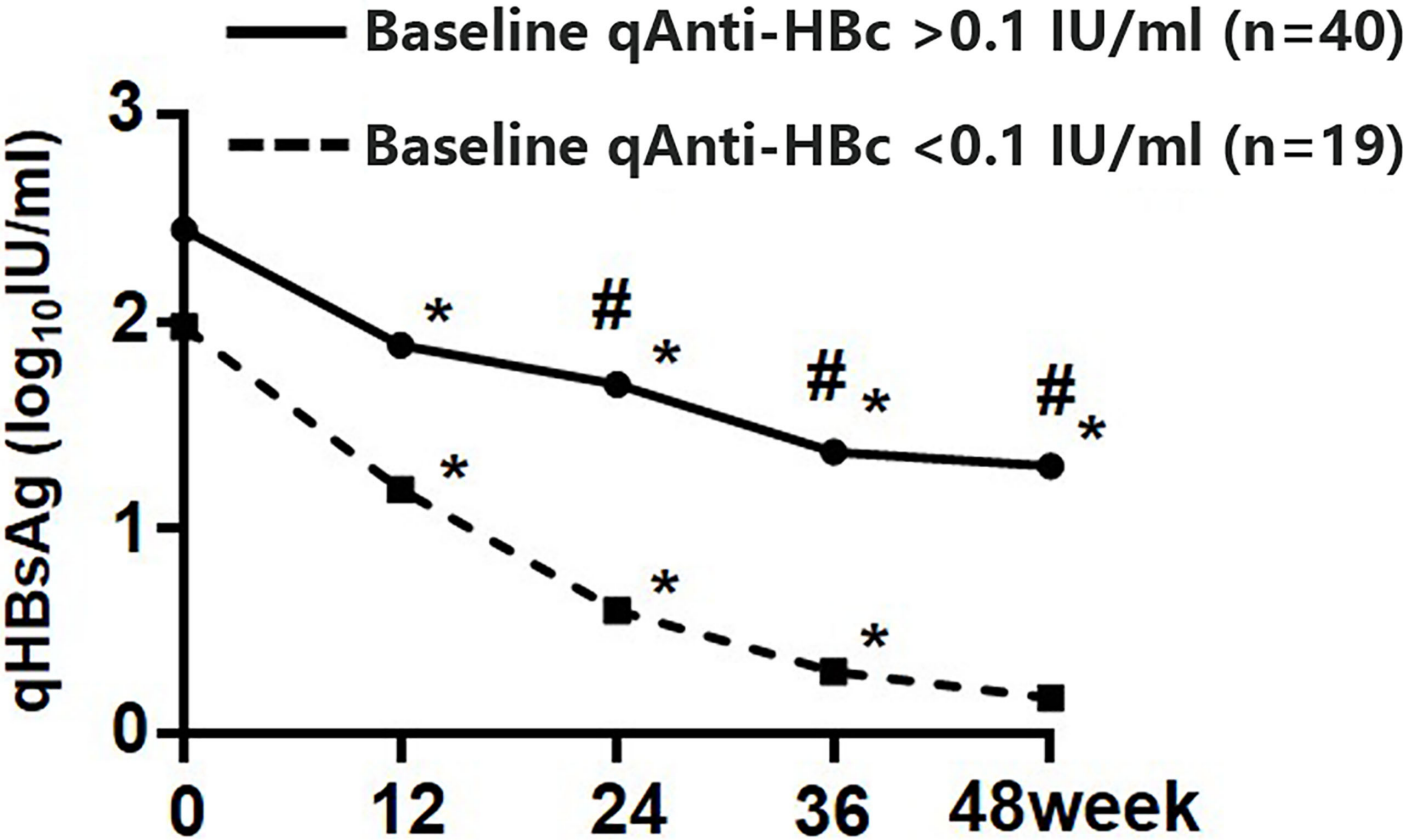
Kinetics of qHBsAg in the add-on group with baseline qAnti-HBc < 0.1 IU/ml and > 0.1 IU/ml. * Comparison in qHBsAg between this and the last time points and *p* < 0.05; # Comparison in qHBsAg between the two subgroups and *p* < 0.05. qHBsAg, quantitative hepatitis B surface antigen; qAnti-HBc, quantitative anti-hepatitis B core antibody.

In those who cleared HBsAg and those who did not, qAnti-HBc levels dropped by 0.00 (0.00, 0.29) log_10_IU/ml and 0.26 (0.00, 0.51) log_10_IU/ml, respectively. Dynamic variation of qHBsAg during 48 weeks of add-on therapy showed that the decrease of qHBsAg at weeks 24, 36, and 48 in patients with baseline qAnti-HBc < 0.1 IU/ml was significantly greater than that in patients with baseline qAnti-HBc > 0.1 IU/ml (**Figure 4**).

**Figure 5:**
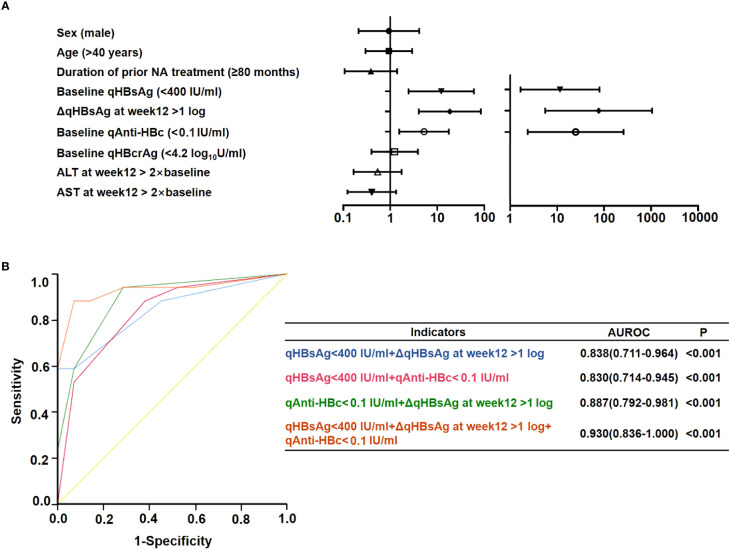
Prediction of HBsAg loss at week 48 in the add-on group. **(A)** Univariate and multivariate logistics regression analysis of factors predicting HBsAg loss at week 48. **(B)** Receiver operating characteristic curve of factors predicting HBsAg loss at week 48. NA, nucleos(t)ide analogs; ALT, alanine aminotransferase; qAnti-HBc, quantitative anti-hepatitis B core antibody; HBsAg, hepatitis B surface antigen; qHBsAg, quantitative HBsAg; qHBcrAg, quantitative hepatitis B core-related antigen; AUROC, area under the receiver operating characteristic curve; Δ, decline in value.

Univariate logistic regression analysis showed that baseline qAnti-HBc < 0.1 IU/ml was a predictor for HBsAg loss after 48 weeks of add-on therapy (OR = 5.238 [1.554–17.653], p = 0.008), and other predictors included baseline HBsAg levels (<400 IU/ml) (OR = 12.187 [2.457–60.453], p = 0.002) and a decline of qHBsAg > 1 log_10_IU/ml at week 12 (OR = 18.571 [4.060–84.955], p < 0.001). Since previous studies found that qAnti-HBc decreased with NA treatment, and the patients had undergone NA therapy for a long time before enrollment, the duration of prior NA therapy was included in the regression analysis, while sex, age, duration of prior NA therapy, early ALT elevation, and baseline HBcrAg levels did not influence HBsAg clearance significantly (**Figure 5A**). Multivariate logistic regression analysis showed that baseline qAnti-HBc < 0.1 IU/ml (OR = 24.83 [2.369–260.220], p = 0.007), baseline HBsAg levels (<400 IU/ml) (OR = 11.516 [1.662–79.779], p = 0.013), and a decline of qHBsAg > 1 log_10_IU/ml at week 12 (OR = 76.673 [5.568–1055.909], p = 0.001) remained independent predictors for HBsAg clearance after 48 weeks of add-on therapy (**Figure 5A**).

### qAnti-HBc combined with qHBsAg can better predict HBsAg clearance under add-on treatment

Area under the ROC curve (AUROC) was used to compare the predictive effectiveness of these independent predictors for HBsAg clearance (**Figure 5B**). The prediction effect of the combination of baseline HBsAg and baseline qAnti-HBc levels was comparable with the combination of baseline HBsAg levels and decline of qHBsAg > 1 log_10_IU/ml at week 12; the AUROCs were 0.830 and 0.838, respectively. Furthermore, the combination of baseline qAnti-HBc levels and decline of qHBsAg > 1 log_10_IU/ml at week 12 had a significantly higher AUROC (0.887). When the three indexes were combined, the AUROC can reach 0.930.

## Discussion

Many studies have demonstrated that low levels of qHBsAg, most considered less than 1,500 IU/ml, are a favorable factor for NA-treated patients receiving add-on or switching therapy of PEG-IFN-α to achieve HBsAg clearance (6–12, 25–27). However, more than half of these patients cannot achieve HBsAg clearance after 1 to 2 years of this combination treatment. More accurate and early prediction of the response to interferon-based therapy could reduce unnecessary use of drugs and avoid their associated side effects. In this study, we found that more than 50% of NA-treated patients with baseline qAnti-HBc <0.1 IU/ml obtained HBsAg clearance after 48 weeks of add-on therapy, suggesting that qAnti-HBc levels might be used to predict HBsAg clearance.

Anti-HBc is a classical serological marker of HBV infection, comprising anti–HBc IgM and IgG antibodies (28). Anti-HBc IgM is positive at the phase of liver inflammation, but becomes negative in the recovery stage; therefore, it can help to distinguish acute HBV infection or acute exacerbation from quiescent CHB (29). Anti-HBc IgG is a marker of current and previous HBV infection, and may persist for a lifetime. The present study used the widely used double-antigen sandwich method to quantify anti-HBc antibodies (18, 30), and the detection results represent the sum of IgG and IgM antibodies (24). HBcrAg is a combined HBV serum biomarker that consists of three related proteins, including hepatitis B core antigen, hepatitis B e antigen, and p22cr protein encoded by the precore/core gene of HBV (31, 32). HBcrAg levels have generally been identified as closely correlated with serum HBV DNA and intrahepatic cccDNA levels in treatment-naïve CHB patients, and are considered to play a vital role in the evaluation, clinical medication, and prognosis of CHB (21, 33, 34).

Our follow-up study demonstrated that qAnti-HBc and qHBcrAg levels decreased persistently in the add-on group compared with the NA group after enrollment. This difference may be related to the mechanism of action of the drugs: NA inhibit HBV DNA synthesis but cannot block the activity of HBV cccDNA; therefore, their effect on the synthesis of antigen components is weak. IFN, as an innate antiviral cytokine, suppresses viral replication and spread by promoting the expression of various proteins involved in RNA degradation, translational inhibition, and cellular apoptosis (35, 36). IFN is also vital to activate innate immune cells and regulate the adaptive immune response (5, 36). Thus, IFN is able to inhibit the synthesis of various HBV components, and then lead to a further decline in antigen and antibody levels. A recent study had demonstrated that the median levels of anti-HBc IgG were significantly higher in HBV cccDNA-positive occult HBV-infected individuals than in cccDNA-negative anti-HBc-positive individuals (37). Consequently, we speculated that qAnti-HBc levels might reflect the activity of intrahepatic cccDNA transcription and mRNA translation just as qHBcrAg does and could be a predictor of HBsAg clearance. This hypothesis was tentatively confirmed in our follow-up cohort study: logistic regression identified lower baseline qAnti-HBc levels as an independent predictor for HBsAg loss after 48 weeks of add-on therapy. Long-term follow-up studies on inactive HBsAg carriers and HBeAg-negative HBV carriers also support our hypothesis to some extent, since the patients in their cohorts had similarly low levels of viral replication and inconspicuous liver inflammation to ours, and they found that patients with low qAnti-HBc levels were more likely to have spontaneous HBsAg and HBV DNA clearance (38, 39).

However, in the studies of treatment-naïve patients with CHB, the predictive role of qAnti-HBc levels seems to be opposite to the above results: Higher baseline qAnti-HBc levels were associated with a higher rate of HBeAg seroconversion or HBsAg loss in patients who were treated initially with either Peg-IFN-α or NA (16, 18, 20), and higher qAnti-HBc levels before treatment and at drug withdrawal were also related to a lower rate of viral recurrence (40). A recent study reports that a higher pretransplantation qAnti-HBc levels corresponded with sustained HBsAg loss after liver transplantation (41). Notably, the patients enrolled in these studies were mostly in the stage of hepatic inflammatory activity with markedly elevated levels of ALT and virus replication. These studies also found that qAnti-HBc levels correlated positively with ALT levels (16), and patients with ALT > two times the upper limit of normal had the highest rate of HBeAg seroconversion (17). These results suggest that a different background of liver inflammation before and after antiviral treatment might explain the completely opposite predictive effect of qAnti-HBc levels. Another possible reason is the different qAnti-HBc antibody composition in different liver inflammatory phases. The total anti-HBc antibody (IgM and IgG added together) and IgM alone levels correlated positively with ALT (38). However, high levels of anti-HBc IgM can only be detected in patients with active liver inflammation and were found at extremely low levels in inactive HBsAg carriers and in patients who achieved a complete virological response to antiviral therapy (38). Based on the above research, we speculated that anti-HBc IgM is the main component under conditions of obvious liver inflammation, whereas anti-HBc IgG is the main component in the quiescent state. Consequently, the skewed composition of qAnti-HBc might be a direct cause of its contrasting predictive effect in different types of patients, and the predictive power of IgG antibodies may be masked in certain cases. The results from a retrospective study using qualitative methods to detect anti-HBc IgG to some extent support our hypothesis and show that low levels of anti-HBc IgG prior to antiviral therapy were associated with HBsAg clearance in patients with NA-induced HBeAg seroclearance (42). Moreover, varying levels of virus replication in treated and untreated patients might also affect qAnti-HBc’s predictive effect.

Consistent with Huang’s findings (43), qHBcrAg decline during treatment period was observed in both patients with and without HBsAg clearance in this study (data not shown), and neither study found that baseline qHBcrAg had a predictive effect on HBsAg clearance. What is inconsistent between the two studies is that Huang’s study found that qHBcrAg decline at week 24 can predict HBsAg clearance at the end of treatment. The reason for these differences may be related to the number of patients enrolled, duration of prior NA therapy, baseline qHBsAg and HBV DNA levels, and treatment course of PEG-IFN-α.

This study had limitations, such as a small sample size, short treatment time, and lack of long-term follow–up. Additionally, the proportion of IgM and IgG in qAnti-HBc was not detected. Therefore, prospective longitudinal studies with larger sample sizes are needed to further demonstrate the prediction role of qAnti-HBc, and to reveal the underlying immune mechanisms.

In summary, our study provides evidence for the first time that the baseline qAnti–HBc levels may predict HBsAg clearance in NA-suppressed patients with CHB who receive sequential combination therapy with PEG-IFN-α. However, the predictive power of qAnti-HBc alone still fails to exceed that of qHBsAg. Hence, a combination of the baseline qAnti-HBc and qHBsAg levels is a better strategy to guide clinical practice.

## Data availability statement

The raw data supporting the conclusions of this article will be made available by the authors, without undue reservation

## Ethics statement

The studies involving human participants were reviewed and approved by the Ethics Committee of the Fifth Medical Center of Chinese PLA General Hospital. The patients/participants provided their written informed consent to participate in this study.

## Author contributions

JF contributed to conception and design of the study. W-XW and J-YL performed the statistical analysis. W-XW wrote the first draft of the manuscript. RJ, F-SW, and JF wrote sections of the manuscript. All authors contributed to manuscript revision, read, and approved the submitted version.

## Funding

This study was supported in part by grants from the Capital Health Research and Development of Special (2020-1-2181, 2016-2-5031), Capital clinical diagnosis and treatment technology research and transformation application project (Z211100002921059), the National Science and Technology Major Project (2017ZX10202201), the National Natural Science Foundation of China (81572462, 81803299, and 81900537), and the Innovation Groups of the National Natural Science Foundation of China (81721002).

## Acknowledgments

The authors would like to thank Professor George K.K. Lau for making significant suggestions for the article and are grateful to all the participants.

## Conflict of interest

The authors declare that the research was conducted in the absence of any commercial or financial relationships that could be construed as a potential conflict of interest.

## Publisher’s note

All claims expressed in this article are solely those of the authors and do not necessarily represent those of their affiliated organizations, or those of the publisher, the editors and the reviewers. Any product that may be evaluated in this article, or claim that may be made by its manufacturer, is not guaranteed or endorsed by the publisher.

## Abbreviations

Anti-HBs, antibody to hepatitis B surface antigen; CHB, chronic hepatitis B; ETV, entecavir; HBcrAg, hepatitis B core-related antigen; HBeAb, hepatitis B e antibody; HBV, hepatitis B virus; HCC, hepatocellular carcinoma; HBcrAg, hepatitis B core-related antigen; HBeAg, hepatitis B e antigen; HBsAb, hepatitis B surface antibody; HBsAg, hepatitis B surface antigen; IFN, interferon; LLD, lower limit of detection; NA, nucleos(t)ide analogs; PEG-IFN-α, pegylated-interferon-alpha; qAnti-HBc, quantitative anti-hepatitis B core antibody; qHBcrAg, quantitative hepatitis B core-related antigen; qHBsAg, quantitative HBsAg; TDF, tenofovir disoproxil fumarate.
